# Informing drivers about events and their location in Variable Message Signs: effects of words versus pictograms

**DOI:** 10.3389/fpsyg.2026.1775997

**Published:** 2026-06-03

**Authors:** Ana Hernando, Pilar Tejero, Javier Roca, Antonio Lucas-Alba

**Affiliations:** 1 ERA/Department of Psychology and Sociology, University of Zaragoza, Teruel, Spain; 2 ERI-Lectura/Department of Basic Psychology, University of Valencia, Valencia, Spain; 3 ERI-Lectura/Department of Developmental and Educational Psychology, University of Valencia, Valencia, Spain

**Keywords:** drivers’ comprehension, experimental design, message understanding, traffic signage, visual information

## Abstract

With advancing road technologies and growing traffic complexity, a major challenge is to inform drivers through clear and concise messages. This study examines how drivers understand Variable Message Signs indicating the location of an event relative to a city. Four message designs were tested, resulting from two crossed factors: the event category was informed by either a word or a pictogram, and the location of the event was indicated by either a word or an arrow. Twenty-four drivers were asked to identify the message as quickly and accurately as possible. Compared to text: (1) for the event, pictograms led to faster (*M_*pictogram*_* = 6112 ms; *M*_*text*_ = 6338 ms) and more accurate (*R_*pictogram*_* = 1.00; *R*_*text*_ = 0.99) responses; (2) for the location, the arrow resulted in worse accuracy (*R*_*arrow*_ = 0.98; *R*_*text*_ = 0.99) and response time (*M_*arrow*_* = 6261 ms; *M*_*text*_ = 6189 ms). Text-pictogram combinations may constitute a functional strategy toward the harmonization and internationalization of road signage, although the effectiveness of specific combinations likely depends on how clearly each element conveys its meaning.

## 1 Introduction

Technological developments contribute to enhance communication with drivers, enabling novel and more efficient signage possibilities. However, it also brings more challenges to address, as it contributes to a more diverse and complex traffic environment. For example, even though comprehension is considered a critical factor in signage efficacy (Zhang and Chan, 2013), previous studies have shown that current design practices can still be improved by following different ergonomic principles (Bañares et al., 2018; Ishartomo et al., 2020; Shinar and Vogelzang, 2013).

Traffic signs can use different formats: text, pictograms, or both (Horberry et al., 2004; WERD/DERD, 2000). But which format is most effective? This debate is still ongoing regarding both fixed and variable signage. On the one hand, crossborder mobility continues to rise, highlighting the increasing relevance of internationalization, and in this scenario, pictograms hold an advantage over text (Dewar and Pronin, 2023). Moreover, pictograms (when correctly designed) would present advantages regarding legibility distance, especially in bad visibility conditions or specific population groups (Dewar and Pronin, 2023; Ells and Dewar, 1979; Horberry et al., 2004). Due to their potential advantages and widespread use, numerous studies have investigated from an ergonomic perspective how to enhance pictorial traffic signs (Bañares et al., 2018; Ben-Bassat et al., 2019, 2021; Jamson and Mrozek, 2017). However, the absence of text entails other costs. For instance, new or unfamiliar pictograms usually face more comprehension problems compared to text messages (Ben-Bassat and Shinar, 2006). In fact, some studies have demonstrated the superiority of text messages, both in accuracy and response time (Shinar and Vogelzang, 2013). The study by Oh et al. (2024) also reported an advantage for text-based messages, compared with graphic-only designs. However, these authors noted that almost all participants were native speakers and that the static presentation of the stimuli does not account for the greater legibility distance that typically characterizes graphic traffic signs. Also, not all concepts can be appropriately translated into a pictorial representation (Dewar and Pronin, 2023).

This issue has also been investigated focusing on Variable Message Signs (VMS; see Wu et al. (2024) for a recent systematic review). For example, a widespread type of VMS in Europe divides the matrix into two columns: one for the pictogram and the other for the alphanumeric text (Ellenberg and Fabre, 1995). These devices can display real-time, traffic-relevant information, such as regulatory, warning, and informative messages (WERD/DERD, 2000). On this area, some studies have found results consistent with the general recommendation of displaying pictorial information (Arbaiza and Lucas-Alba, 2012). Moreover, different population groups, such cross-border drivers (Wang et al., 2007), older drivers (Ben-Bassat and Shinar, 2015; Wang et al., 2007) or adults with dyslexia (Roca et al., 2018b), can benefit from messages displayed on VMS that use pictograms or graphics. However, contrasting results have also been reported. Roca et al. (2018a) used a simulator in which participants had to drive and identify a single word or a single pictogram on VMS they encountered along the route. The results showed that the response distance in words was shorter than in pictograms, so they concluded that design aspects (e.g., VMS aspect ratio or the relative size of words and pictograms) can have an impact on the acquisition of the information provided by VMS.

Combining text and pictograms in the VMS might be an adequate strategy. According to the *Framework for harmonised Implementation of VMS in Europe* (WERD/DERD, 2000, p. 14), “informative messages should be presented preferably in a pictographic way,” and “textual parts of informative message should be brief and unambiguous.” So, combining both formats is allowed, but how these elements should be effectively combined remains unclear. The majority of studies on this subject have examined displays in which either text or pictograms are used to specify the meaning of the other ones (Dewar and Pronin, 2023; Ilkhani et al., 2023), or displays in which pictograms and words are redundant (i.e., pictograms and words provide the same information). For example, Wang et al. (2007) compared text messages displayed on VMS to the same messages accompanied by a pictogram on the left side. They showed that 94% of participants preferred the graphic-aided messages, and the response time was shorter for these messages compared to the text-only versions, especially among non-native-English speaking drivers. Also, Zhao et al. (2019) found that the drivers’ preferences depended on weather conditions, with graph-only messages being preferred under foggy conditions, while text or text-graph combinations were favored under normal visibility.

To our knowledge, there is limited evidence on the effectiveness of combining text and pictogram without redundancy, i.e. the pictogram and the text showed different information, particularly in messages displayed in VMS. Fancello et al. (2021) provided descriptive results suggesting that non-redundant combined messages were less understood compared to text-only messages, or redundant combined messages. However, in this particular study, the number of responses collected was limited, and the messages included pictograms surrounded by a red triangle, which could be detrimental for pictogram identification (Roca et al., 2018a). Therefore, the present study aims to contribute further evidence by examining several non-redundant message formats within a controlled experimental task.

The present study focused on the comprehension of VMS displays informing drivers about a given traffic event and its location, e.g., “ROADWORKS BEFORE COSLADA.” We conducted an experiment to study the differences in comprehension depending on the use of visual items (pictograms, arrows) or text (words) for conveying two specific elements of the message: the event category, and the event location. The event category (e.g., roadworks) was informed by a single word or the official pictogram (Legislación consolidada, 2003). We expected that pictograms would be more effective than text in conveying information about the event. As for the event location, this study focused on the case of the VMS informing about events at a long distance from the VMS. Due to these particularities—namely, the potential long distance from the VMS and the volatility of such traffic events—using numerical indicators (e.g., kilometers or miles) to convey the event location may not be the optimal solution. In fact, current recommendations for these VMS suggest displaying qualitative location instead, e.g., if the event will be encountered before or after reaching a given city in the route (Arbaiza and Lucas-Alba, 2008; WERD/DERD, 2000). In the present study, we focused on messages indicating that the event was located before a given city, considering previous studies showing that this particular option is easier to understand than those indicating that the event is located after a given city (Hernando et al., 2022). In particular, in half of VMS displays on the experimental condition, the location was informed by the word “before” plus the name of the city. In the other half, instead of the word “before,” the location was informed by an arrow pointing to the name of the city. Due to its versatility and compatibility with the VMS matrix, and its deictic characteristics (equivalent to a pointing finger; Eco, 1995), the arrow is the “diagram” (Kurata and Egenhofer, 2005) or “graphical device” (Tversky et al., 2000) most suitable for indicating relative location, in line with other traffic signs (e.g., advanced directional signs). However, to our knowledge, there is limited evidence directly comparing the effectiveness of an arrow and text for conveying location information or examining how different formats interact when combined within the same message. Therefore, the present study explores the comparison between arrow and text for location, as well as the effectiveness of combining these formats. In short, the present study examined the effects of the format used for the event category (a word or a pictogram) and the format used for the event location (the word *before* or an arrow) on the comprehension of messages in VMS.

## 2 Materials and methods

### 2.1 Participants

A sample of 24 drivers (18 women) completed the experimental session. The average age was 28.13 years (*SD* = 6.38; *MIN* = 18; *MAX* = 43). All of them had completed or were pursuing university studies, and held a B-category driving license (which allows driving motor vehicles with a maximum authorized mass of up to 3,500 kilograms and transporting no more than eight passengers plus the driver). The average driving experience was 8.27 years (*SD* = 6.35; *MIN* = 0.05; *MAX* = 24.53).

### 2.2 Procedure

To recruit potential participants, information about the study was sent to students and staff at the University of Valencia, and also, several announcements were placed in different parts of the Faculty of Psychology and Speech Therapy. Potential candidates voluntarily agreed to participate and no economic compensation was offered for participation. Informed consent was obtained from each participant. This research complied with the tenets of the Declaration of Helsinki and was approved by the Ethics Committee of Research in Humans of the University of Valencia.

First, the experimenter exposed a brief explanation regarding the experiment. The basic disinfection protocol was followed in the context of COVID-19. Before starting the experimental task, each participant read and signed the informed consent form. Once the participant was settled into the computer seat, the experimenter started the task on the computer, which included all the instructions necessary to complete it. First, the objective was explained and he or she answered sociodemographic questions. Next, the context of the task was explained. The participant practiced for 20 trials responding to each message set separately (8 from the target trial set and 4 from each catch-trial set; see Task) and then completed 24 additional practice trials that included examples from all sets. The presentation order of practice trials was random. After each response, feedback (correct/incorrect) was displayed on the screen to the participant. The experimenter remained in the room to clarify possible doubts. Once practice trials were completed, the experimental task began and the experimenter remained in the adjunct room. No feedback was given to the participant during the experimental task. The task consisted of 192 trials in total, presented in random order to the participant. To give the opportunity to rest, the task was divided in three blocks. First, 64 trials and then it appeared a message in the screen informing that they could rest briefly. When they were prepared to continue, the experimenter resumed the task. After 64 trials, the same message was shown.

### 2.3 Task

The task was contextualized within an imaginary route passing through the Spanish cities of Alcalá, Torrejón, and Coslada. Participants were told that a series of VMS would display relevant events (roadworks, congestion, snow, or wind) occurring *before* one of the cities in the route. In addition to these critical cities and events, additional VMS messages were introduced to lower response predictability and prevent participants from developing automatic response patterns. These messages included some cities outside the route (Alcañiz, Ávila, Tortosa, Terrasa, Cornellà, or Córdoba), non-relevant informative events (radar, airport, factory, or monument), or events located *after* the cities in the route as “catch trials” (see below for further information on experimental and catch trials). In particular, we chose those alternative cities outside the route because (a) they are geographically far away from the real route, (b) they share the first letter with one of the cities included in the route, and (c) they have same number of syllables as the cities in the route.

**FIGURE 1:**
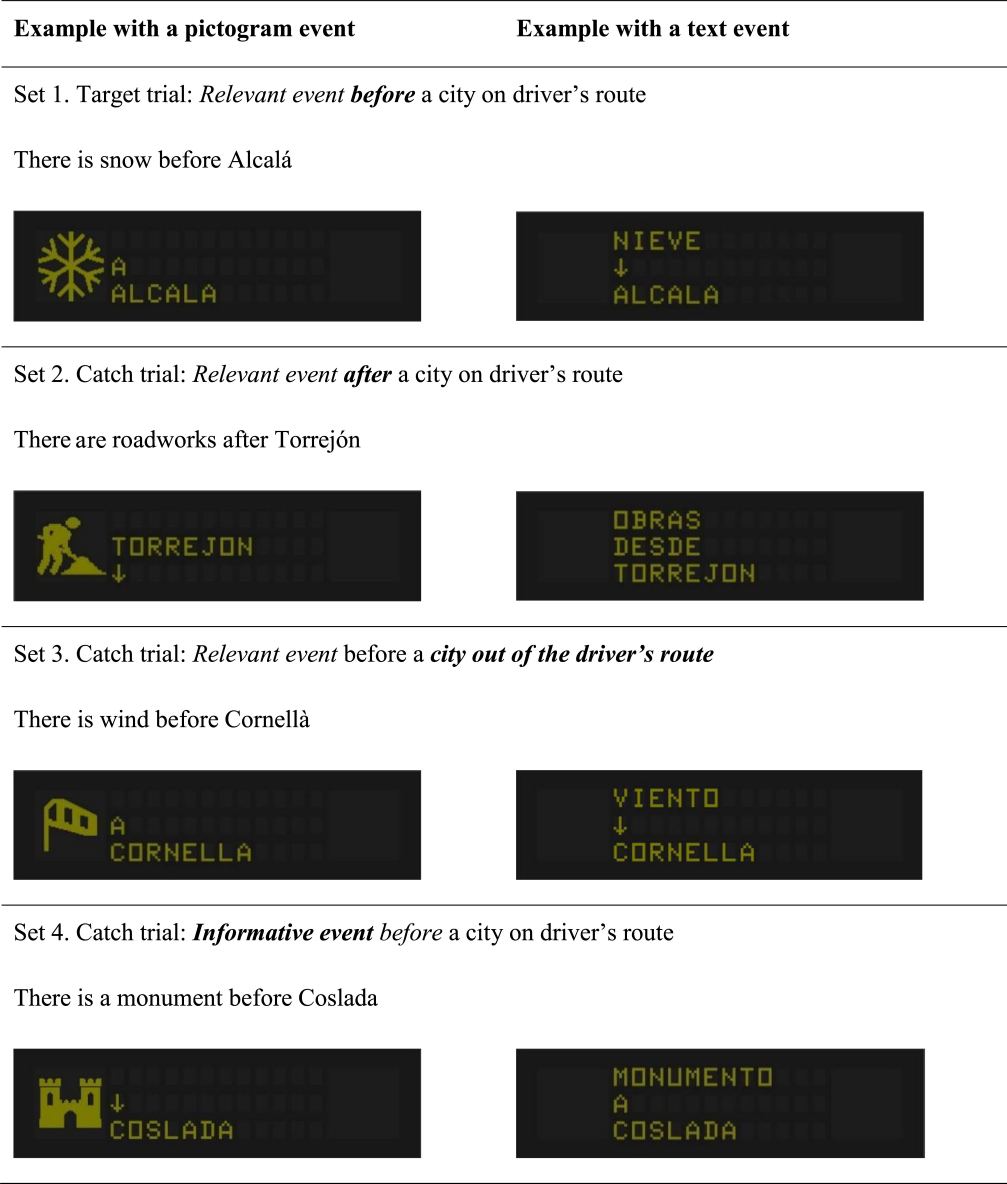
A stimulus example of the four sets of messages included in the experimental task. In the messages on the VMS, city names were written without accents, following the common practice in Spain.

The participants were asked to indicate, as soon as they could respond correctly, which of the following options applied for each VMS presented (Figure 1):

(1) The message informed about a relevant event happening *before* a city in the route. For these messages, the correct response was to press the key labeled “a” (which mapped to the “s” key on the keyboard), meaning “antes” (the Spanish word for *before*).
(2) The message informed about a relevant event happening *after* a city in the route. For these messages, the correct response was to press the key labeled “d” (which mapped to the “l” key on the keyboard), for “después” (the Spanish word for *after*).
(3) The message informed about a relevant event located before a city *outside the route*, or an *informative* event before a city in the route. For all these messages, the correct response was to press the space bar.

To summarize, the trials presenting the messages described in point 1 above (i.e., a relevant event happening before a city in the route) were the actual target trials of the experiment. Participants did not know which trials were the target of the study.

### 2.4 Stimuli and apparatus

The experiment was carried out in a light- and sound-attenuated laboratory at the Faculty of Psychology and Speech Therapy of the University of Valencia. A computer with a 47 × 26.80 cm monitor (1,920 × 1,080-pixel resolution) and a keyboard were used to perform the task. To control the distance from the screen to the participants, a chinrest was located at 57 cm from the screen.

**FIGURE 2:**
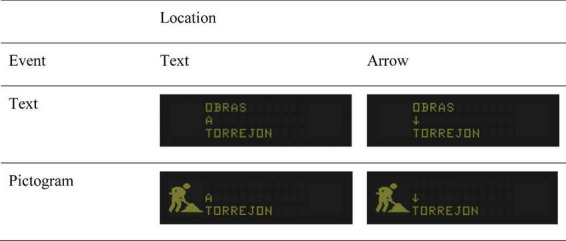
A stimulus example of each condition included in the experimental design. The meaning of the four messages was “There are roadworks before Torrejón.” The category of the event was indicated by either text (e.g. the Spanish word “*obras*,” meaning *roadworks*) or the corresponding pictogram. The location of the event relative to the city (in the example, Torrejón) was indicated by either text (the Spanish preposition “*a*,” meaning *to*) or an arrow.

The stimuli used in this study were designed to approximate the requirements set forth in the 1968 Vienna Convention and EN 12966. It should be noted, however, that these standards represent a consensus-based framework for industrial uniformity across Europe. They do not necessarily constitute the empirically “ideal” configuration for driver communication, but rather the current legal and technical benchmark against which new empirical data—such as the results of this study—should be evaluated. Stimuli were VMS models, similar to the VMS used in Spanish motorways, showing messages about an event and its qualitative location. To create the stimuli, we used Blender v.2.79b, a free and open-source 3D creation software. First, we created a 3D model of the VMS, following the specifications of the standard norm UNE-EN 12966 for the VMS dimensions, spacing, colors, and other design features (Asociación Española de Normalización y Certificación, 2015). This model could display three 12-character text lines (including arrows) and also two pictograms on left and right sides of the VMS. Each character was represented in a 5 × 7 dot matrix (3,910 × 1,270 millimeters), with a dot size of 45.6 millimeters, so the character height was 320 millimeters. Each pictogram was represented in a 64 × 64 dot matrix (1,270 × 1,270 millimeters), with a dot size of 19.8 millimeters. In the present study, all stimuli included some information on the area for text, and some stimuli also showed a pictogram on the left area of the VMS. According to Spanish regulations (Arbaiza and Lucas-Alba, 2008), when the event is located far from the VMS, warning of danger is not recommended (the study pictograms were not surrounded by a red triangle; see Hartz and Schmidt, 2005, for similar use in Germany). The right-side pictogram area was empty in all stimuli. The contents of the VMS were created for the experiment purposes, using four different designs (see the examples in Figure 2): (a) text-text messages, b) arrow-text messages, (c) pictogram-text messages, and (d) pictogram-arrow messages.

Stimulus presentation was dynamic: in each trial, the participant watched a short video of the VMS, created with Blender. In the video, the VMS expanded radially, to simulate that the participant was approaching to the VMS and passed under the gantry. The messages were not legible from the start, ensuring that the participants could not respond immediately. The video lasted a maximum time of 10 seconds, stopping when the participant responded before the video ended. An example of the video is available at https://osf.io/zdwv2/?view_only=00721b3e37224c0594954102950dba9a.

### 2.5 Design and analysis

There were 96 target trials (the message informed about a relevant event happening *before* a city in the route): 24 trials per condition resulting from the crossing of the two formats of the event category (pictogram or text), and the two formats of the event location (arrow or text). To achieve the necessary number of trials per condition, we combined the four relevant events (roadworks, congestion, snow, or wind), the three cities in the route (Alcalá, Torrejón, and Coslada), and two repetitions. The remaining trials (described in points 2 and 3 of the 2.3. *Task* section) were *catch trials*, and were included to reduce the predictability of correct responses and prevent a potential automation of the task execution. Catch trials included: 32 trials presenting messages about a relevant event *after* a city of the route, 32 presenting a relevant event *outside* the route, and 32 presenting an *informative* event. All other relevant factors (such as the format of the event category, and the format of the event location, event, or city) were balanced within each of these three groups of catch-trials. In summary, an equal number of target and catch trials (96 each) were presented.

Data analysis only included the target trials. Comprehension was assessed using two complementary measures: response accuracy (ratio of correct responses per participant and condition), and response time (the time that elapsed between the stimulus onset and the participant’s button press, in milliseconds).

We performed a generalized linear mixed model (GLMM) with a binomial distribution (logit), considering response accuracy at the trial level as the dependent variable; format for the event category (event: text/pictogram) and format for the event location (location: text/arrow) as fixed effects variables; and random intercept for each participant. An alternative model including random slopes was tested, accuracy ∼ event * location + (1 + event * location| participant), but failed to converge successfully. These models were analyzed with lme4 package v.1.1–29 (Bates et al., 2015) implemented in R 4.2.0 (R Core Team, 2021). Response time data analysis was performed using IBM SPSS Statistics v26, and only correctly responded trials were included in the analysis. Additionally, trials with extreme values (i.e., those deviating by more than ± 3 standard deviations from the mean for each participant and condition) were removed from analysis, comprising 23% of the trials. A 2 × 2 repeated measures ANOVA model was used: Event (pictogram/text) and Location (arrow/text). Both factors were manipulated as within-participant factors. For exploratory purposes, driving experience was included as a covariate in time response analysis. On both cases, the level of significance was set at *p* < 0.05.

## 3 Results

Table 1 shows average results of time (milliseconds) and response accuracy (percentage of correct responses per participant and condition) by the four message conditions used in the experiment, resulting from the combination of the two factors: Event (pictogram/text) and Location (arrow/text).

**TABLE 1:** Summary of results (Mean, M; Rate, R; Standard Error, SE; and 95% confidence interval, 95% CI) by VMS message condition (Event: Pictogram/Text × Location: Arrow/Text).

Conditions		Response time (ms)			Response accuracy		
Event	Location	*M*	*SE*	95% CI	R	SE	95% CI
Pictogram	Text	6089	89	5904–6274	0.99	0.10	0.98–1.00
Pictogram	Arrow	6134	102	5923–6346	0.94	0.23	0.92–0.96
Text	Text	6289	95	6092–6487	0.97	0.17	0.96–0.98
Text	Arrow	6388	101	6177–6598	0.94	0.23	0.92–0.96

**FIGURE 3:**
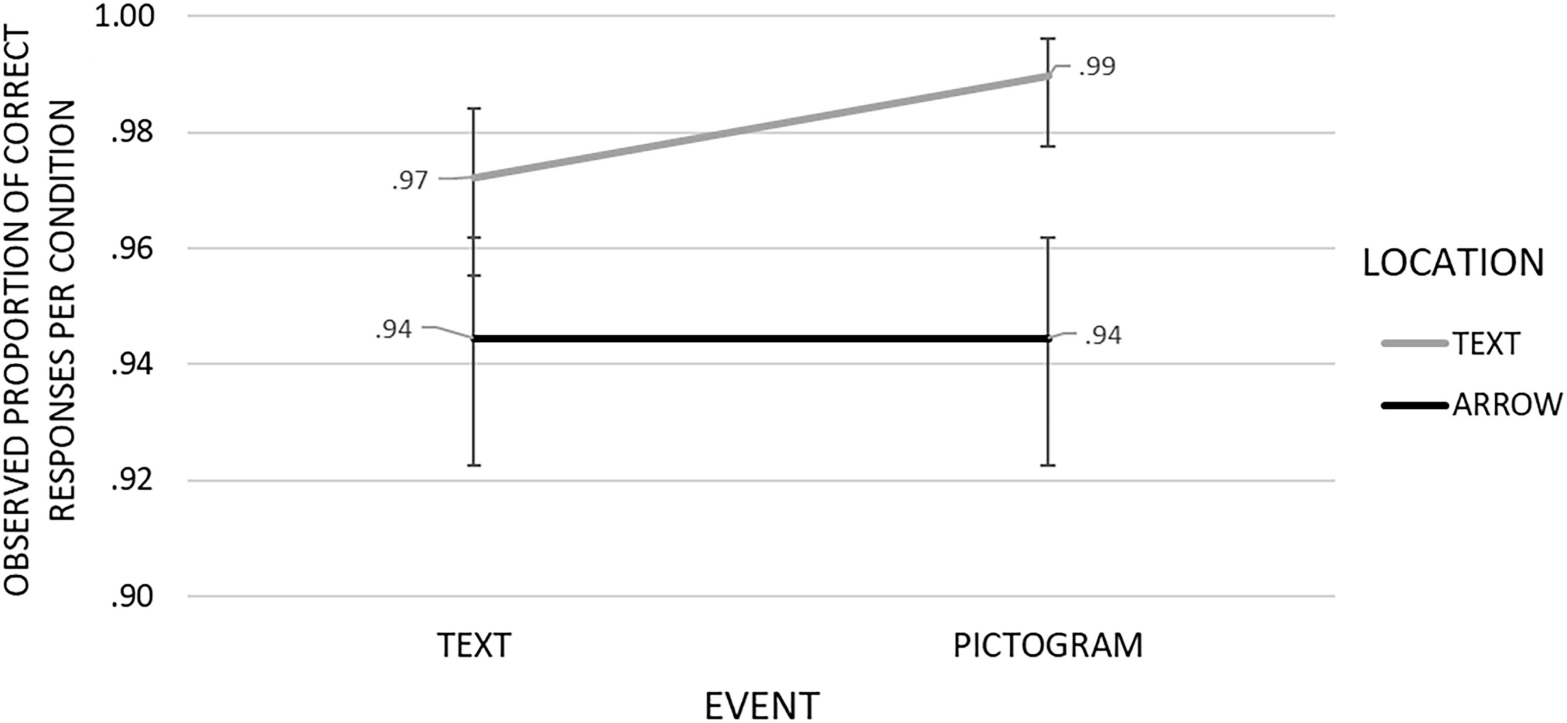
Response accuracy by VMS message condition (Event: Pictogram/Text × Location: Arrow/Text). Error bars show the 95% confidence interval.

Regarding the response accuracy, although all message formats reached a high accuracy rate (R), responses were more accurate when the VMS used a pictogram for the event [*R* = 1.00; CI = (0.99, 1.00)], compared to text [*R* = 0.99; CI (confidence interval) = (0.98, 1.00)], *z* = 2.125, *p* = 0.034; *OR* (odd ratio) = 2.85. Also, responses were less accurate when the location was informed by the arrow (*R* = 0.98; CI = [0.95, 0.99)], compared to text (*R* = 0.99; CI = [0.98, 1.00]), *z* = -2.438, *p* = 0.015; *OR* = 0.45. The interaction was not significant, *z* = -1.855, *p* = 0.064; *OR* = 0.35 (Figure 3).

**FIGURE 4:**
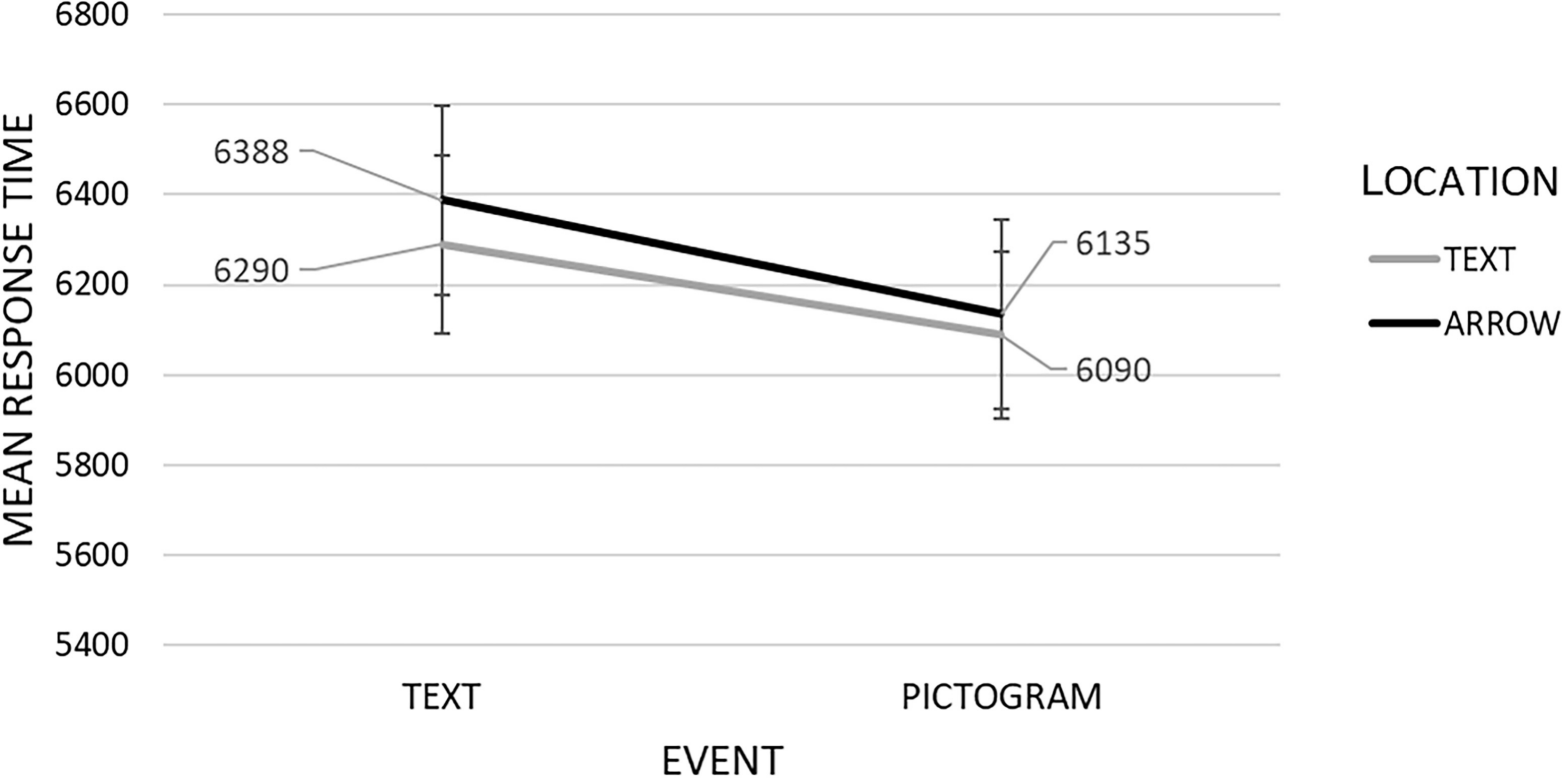
Average response time (ms) by VMS message condition (Event: Pictogram/Text x Location: Arrow/Text). Error bars show the 95% confidence interval.

Regarding response time, we observed the main effect of the factor Event, *F*(1, 23) = 58.011, *p* < 0.001; η_*p*_^2^ = 0.716. On average, participants answered faster when the VMS used a pictogram to inform about the event [*M* = 6112; CI = (5914, 6309)], compared to text [*M* = 6338; CI = (6138, 6539)]. We also explored the potential effect of driving experience, although it did not show a significant main effect for time response (*p* = 0.890). However, a significant interaction with the factor Event emerged for time response [*F*(1, 22 = 6.673, *p* = 0.017; η_*p*_^2^ = 0.233), suggesting that the advantage of pictograms over text may increase with experience. Also Location showed a significant effect on response time, *F*(1, 23) = 7.94, *p* = 0.010; η_*p*_^2^ = 0.257. Responses to VMS using the arrow [*M* = 6261; CI = (6053, 6469)] were slower than those to VMS using text for the event location [*M* = 6189; CI = (6001, 6377)]. Finally, the interaction between both factors was not significant, *F*(1, 23) = 2.12, *p* = 0.159; η_*p*_^2^ = 0.084 (Figure 4).

## 4 Discussion

This study aimed to examine if the comprehension of a message in a VMS would improve by combining different message formats including non-redundant text or pictograms. Particularly, we tested messages that informed drivers about an event category (by using a word or a pictogram) and its qualitative location (by using a word or an arrow). The results suggested that participants comprehended the message better when the event category was informed by using a pictogram, compared to text (response time was 226 ms slower, response accuracy was 1% higher). However, for the event location, text was more effective compared to an arrow (response time was 72 ms slower, response accuracy was 1% higher). The results showed that drivers are able to respond with a high level of accuracy to messages conveyed through a combination of text and pictograms [response accuracy was 99% for the combination of pictogram (event) and text (location), and 94% for the combination of text (event) and arrow (location)]. More importantly, the findings suggest that the effectiveness of a display in communicating the message depends on which format (pictogram or text) is used for specific components of the message. For example, events are better represented by pictograms than by words, whereas the event location relative to a given place is better conveyed using words than arrows.

Firstly, our results showed that messages that displayed pictograms to communicate the event category were associated with faster and higher percentage of accurate responses, compared to text. These results are in line with previous studies that advocated an advantage of the pictogram over text (Arbaiza and Lucas-Alba, 2012; Dewar and Pronin, 2023), while they are in contrast with previous evidence suggesting that simple text messages (i.e., a single-word informing about an event) could be related to higher legibility distance and comprehension as compared to pictograms (Roca et al., 2018a). To this respect, it should be noted that the current study used more complex messages (event + location) than in Roca et al. (2018a)’s study. In addition, due to the informative character of our messages, the red triangle was removed and the pictogram occupied the entire area allowed on the VMS, possibly maximizing the distance at which the pictogram is legible in comparison to Roca et al. (2018b)’s study. However, previous studies showed that, by removing the red triangle, responses related to danger or warning decreased, but not the understanding of the event (Lucas-Alba et al., 2011). Therefore, we did not expect differences in comprehension after removing the red triangle. In sum, our results provide further evidence of the effectiveness of the pictogram to inform about traffic events. Regarding driving experience, an interaction emerged with the element used to convey the event, suggesting that the advantage of pictograms over text in response time may increase with greater driving experience. As this analysis was conducted in an exploratory manner to aid interpretation of this interaction, these findings should be interpreted with caution. Further research is needed to more specifically examine how driving experience may influence the comprehension of different message formats.

Secondly, messages that conveyed the event location by using text showed faster and more correct responses, compared to the arrow. Therefore, the pattern of results observed when informing about the location (better performance with text) is reversed to the results described for the event (better performance with the pictogram). One potential explanation would refer to the versatility of the arrow, since it is a graphic element that can be interpreted differently depending on the surrounding elements, i.e., the disposition of the elements on the VMS (Kurata and Egenhofer, 2005; Tversky et al., 2000). According to Dewar and Pronin (2023), when the direction in which the sign must be read is ambiguous, an arrow can be helpful. Nevertheless, those same physical properties that grant versatility and allow for conveying multiples meanings (causal and functional), may also constitute a source of uncertainty (Kurata and Egenhofer, 2005; Tversky, 2005; Tversky et al., 2000).

Regarding our study, we must remind that the participants were informed about the content of the messages and they were exclusively attending them (i.e., they did not simultaneously drive). Considering this, the arrow still managed to deal with some ambiguity, particularly reflected on response accuracy results. Finally, the text and arrow dimensions are aligned with the actual measurements of the VMS, thereby, they are constrained by the device (the arrow or the text cannot be enlarged). In our case, following the linguistic inertia of text messages (from left to right, top-down; Bergen and Chan, 2005; Spalek and Hammad, 2005), the arrow was also located top-down. However, the case of the qualitative location should be further studied and there are more options that need to be explored. For example, previous studies (Hernando et al., 2022) showed different results in comprehension of VMS messages regarding the direction of the arrow and the disposition of the elements (top-down vs. bottom-up).

As the interaction was not significant in the current study, the reported effects of the pictogram to inform about the event and the text to inform about the location must be considered summative. In consequence, the most successful message combination was formed by pictogram-event and text-location (99% of accuracy response and 6089 ms; see Table 1), while the worst performance was observed in the reversed combination (text-event and arrow-location; 94% of accuracy response and 6388 ms). In contrast with the study by Fancello et al. (2021), these results lead to an interesting finding: combining pictograms and text could be effective to communicate traffic information on VMS, but the best option would depend on the specific elements in the message. Moreover, Wang et al. (2007) also found an advantage in incorporating the pictogram on the left side of the VMS over the text-only messages; although in that case the text message was redundant (both the text and the pictogram conveyed the event category). Our results reflect that non-redundant combined messages might be effective while reducing the length of the message.

### 4.1 Practical applications, limitations and further research

In connection with practical applications on this area, it is important to consider also a cost/benefit perspective of using each format to convey traffic information. Clearly, language remains to be an obstacle when designing signs to enhance comprehension of international drivers and other population groups (Arbaiza and Lucas-Alba, 2012; Shinar and Vogelzang, 2013). Thus, research must consider not only the effectiveness of the format, but also the accessibility of the signage and its elements (in our case: text and pictograms). Given the limited evidence on the efficacy of these complex messages, this study constitutes a fundamental step, starting with an experimental task in a laboratory context.

According to our results, all four combinations exceeded the 90% of correct responses and the maximum difference on time response was around 300 ms. When driving at a speed of 120 km/h, drivers can travel up to 10 meters in 300 ms, whereas the time available to read a VMS message is approximately 4.5 s (Montoro et al., 2004). However, placing these results in their context, it should be noted that these findings have been obtained within a specific task. Firstly, before the experiment, the participants were informed about the content of these messages, and they also completed multiple practice trials, which might limit the potential differences among the combinations on accuracy responses. Future studies could examine whether similar results are replicated in the absence of prior practice. Replicating these findings using different task paradigms could also help determine whether the observed advantage of text or pictograms remains consistent across different task demands. Secondly, the participants did not do anything but perform the comprehension task, so it would be interesting to test these VMS messages in a driving-simulator, improving the ecological validity of these findings. Although response accuracy was very high overall, we still observed significant differences in error rates among the experimental conditions. Therefore, our next step is to verify whether these reported differences would be replicated (or hypothetically maximized) when the experimental task becomes more complex -either by requiring participants to reason about the location of the event or by having them simultaneously drive while performing the comprehension task-. Especially in the first case, future studies could examine how prior and contextual knowledge influences reasoning about the meaning of the messages. In addition, these behavioral indicators might be complemented with empirical evidence about drivers’ actual comprehension of these messages (i.e., comprehension test).

Additionally, in order to enhance the generalizability of the results, further studies should make an extra effort regarding two issues. First, the current study included young drivers (mean age = 28.13), predominantly women with college education. Therefore, further studies should include drivers from different age, educational levels and driving experience ranges, as well as non-native drivers; and aim for a more balanced gender distribution. Also, we presented in the experimental task high-frequent pictograms on the road system. Future research should explore whether the present findings generalize to less effective or poorly designed pictograms, currently included in official catalogs (Ben-Bassat et al., 2019; Shinar and Vogelzang, 2013).

This study has certain methodological limitations. First, while the semantic recognition of the pictograms was validated through our experimental protocol, the stimuli themselves only approximate the physical characteristics defined by the 1968 Vienna Convention and EN 12966. This is because the physical photometry (luminance and contrast) of a computer display cannot replicate the photometric performance of certified road hardware. Thus, future field studies would be required to verify how these findings interact with extreme outdoor lighting conditions. Second, our results do not represent an unambiguous psychophysical assessment of “effectiveness” under standardized conditions. In particular, we did not experimentally control for response criterion (bias) or the speed-accuracy tradeoffs. Therefore, our findings primarily reflect the semantic comprehension of the pictograms rather than a complete analysis of participants’ psychophysical response. Future research should validate these cognitive findings under real-world physical conditions and varying response pressures.

Another important direction concerns the usefulness of redundant text-pictogram combinations -in which both elements convey the same information- when messages involve complex meanings, as in the present case. Future studies should explore the potential benefits of this strategy -for instance, during the implementation of new traffic signs- as well as its possible costs in terms of increased message length and cognitive load (Xu et al., 2020).

## 5 Conclusion

In conclusion, our results suggest that messages displayed on VMS that combine pictograms and text might be an adequate strategy. However, the ability of each format in conveying specific information is crucial for the overall message to be effective. Consistent with the recommendations of international sign conventions (e.g., the 1968 Vienna Convention), our results regarding the event category confirm the functionality of using pictograms over text. Regarding location, although limited to our particular case, it seems more complex to replace language with elements like the arrow. In addition to being effective, efforts must be made to ensure that signage becomes increasingly accessible. Therefore, practical implications of using text or pictograms must be considered when revising traffic sign design guidelines and recommendations. Further research will be useful to examine effective strategies for combining these formats in conveying messages on VMS.

## Data Availability

The datasets generated for this study can be found in the OSF repository (https://osf.io/zdwv2/?view_only=00721b3e37224c0594954102950dba9a).
